# Can Immersive Sound Therapy Counteract Neurodegeneration by Enhancing Glymphatic Clearance? Comment on Sachdeva et al. Effects of Sound Interventions on the Permeability of the Blood–Brain Barrier and Meningeal Lymphatic Clearance. *Brain Sci.* 2022, *12*, 742

**DOI:** 10.3390/brainsci13010098

**Published:** 2023-01-04

**Authors:** Peter Wostyn, Piet Goddaer

**Affiliations:** 1 Department of Psychiatry, PC Sint-Amandus, 8730 Beernem, Belgium; 2 Studio Ozark Henry, 8670 Wulpen, Belgium

**Keywords:** Alzheimer’s disease, glymphatic system, immersive sound, meditation, neurodegenerative disorders, slow-wave activity

## Abstract

We would like to congratulate Sachdeva and colleagues for establishing an informative review regarding the effects of music/sound exposure on blood–brain barrier permeability and meningeal lymphatic/glymphatic clearance, and would appreciate the opportunity to make a comment. The review by Sachdeva and colleagues documents the beneficial effects of sound interventions on blood–brain barrier permeability and the activity of the meningeal lymphatic/glymphatic system. The authors further note that sound interventions may have the potential to reduce the accumulation of amyloid-β within the brain in Alzheimer’s disease through improved meningeal lymphatic/glymphatic clearance. The authors also nicely discuss evidence that music influences sleep quality, which may facilitate glymphatic solute clearance as a result of an increase in the interstitial space, which results in reduced resistance to fluid transport. We fully agree with this notion, since we recently hypothesized that immersive sound therapy may be an innovative approach to reduce the individual risk of developing neurodegenerative diseases, such as Alzheimer’s disease, by inducing EEG slow-wave delta oscillations (which characterize deep sleep), thereby promoting glymphatic clearance.

We read with great interest and enthusiasm the article by Sachdeva et al. [1] entitled ‘Effects of sound interventions on the permeability of the blood–brain barrier and meningeal lymphatic clearance’ published recently in *Brain Sciences.* We would like to congratulate the authors for establishing an informative review regarding the effects of music/sound exposure on blood–brain barrier (BBB) permeability and meningeal lymphatic/glymphatic clearance, and would appreciate the opportunity to make a comment.

The review by Sachdeva et al. [1] documents the beneficial effects of sound interventions on BBB permeability and the activity of the meningeal lymphatic/glymphatic system. The authors further note that sound interventions may have the potential to reduce the accumulation of amyloid-β (Aβ) within the brain in Alzheimer’s disease (AD) through improved meningeal lymphatic/glymphatic clearance. The authors also nicely discuss evidence that music influences sleep quality, which may facilitate glymphatic solute clearance as a result of an increase in the interstitial space, which results in reduced resistance to fluid transport. As discussed below, we fully agree with this notion, since we recently hypothesized that immersive sound therapy may be an innovative approach to reduce the individual risk of developing neurodegenerative diseases, such as AD, by inducing electroencephalographic (EEG) slow-wave delta oscillations (which characterize deep sleep), thereby promoting glymphatic clearance [2].

The glymphatic system, first described in 2012, is a brain-wide perivascular pathway for cerebrospinal fluid (CSF)–interstitial fluid (ISF) exchange that enables the clearance of brain metabolic waste products, including Aβ [3]. Senile plaques composed of Aβ are one of the pathologic hallmarks of AD. The glymphatic system consists of a periarterial CSF influx pathway, a perivenous ISF efflux pathway, and a trans-parenchymal pathway that is dependent upon astroglial aquaporin-4 water transport [3,4]. Impairment in the function of the glymphatic system has been demonstrated in the aging brain and has been implicated in neurodegenerative diseases such as AD [5,6,7]. Therefore, discovering strategies for improving glymphatic clearance could have significant preventive and therapeutic implications for brain health. Based on the considerations below, we speculate that meditation-based approaches, such as immersive sound meditation, may have the potential to counteract neurodegeneration by enhancing the efficiency of glymphatic solute clearance [2].

An intriguing finding is that the glymphatic system is suppressed during wakefulness, while highly active during sleep, particularly non-REM slow-wave sleep [8]. Slow-wave sleep or deep sleep is characterized by EEG slow (delta) waves, which are high amplitude 0.5–4 Hz brain waves [8]. Experimental evidence from mice has demonstrated that slow-wave sleep is associated with increased glymphatic CSF influx and a doubling in the glymphatic clearance of Aβ [9]. This appears to be attributable to a 60% increase in the interstitial space during sleep, which presumably reduces tissue resistance to interstitial fluid flux [9]. Further analysis showed that locus coeruleus-derived norepinephrine might be responsible for suppressing the glymphatic system in the wakened state as a result of contraction of the interstitial space, restricting the movement of ISF [9].

An intriguing question is whether slow-wave activity can be enhanced through meditation-based approaches. Studies investigating the EEG patterns associated with meditation seem to support this possibility. Indeed, several studies have found increased delta power across a variety of meditation practices [10,11]. Based on what is known about the glymphatic system and meditation, we hypothesized that meditation-based approaches could be neuroprotective by enhancing glymphatic clearance via inducing slow-wave delta oscillations [2]. More specifically, we proposed immersive sound meditation as an attractive candidate for modulating glymphatic function [2]. Immersive sound is a realistic, multi-dimensional sound experience where the listener is fully enveloped by sounds, which are perceived as coming from an infinite number of points. Interestingly, immersive sound can also be used as a tool for meditation. Indeed, sounds can be specially designed to suit meditation-specific characteristics. The advantage of such a meditation-based approach is that it is a safe, non-invasive, low-cost method that is easy to apply and does not require a great deal of discipline from the meditator. As noted above, increased EEG delta waves have been documented in relation to meditation practices. Moreover, various types of external stimuli, including auditory stimuli and especially music, can influence the bioelectrical brainwave activity [12]. From this point of view, immersive sound meditation could be applied to modify the bioelectrical activity of the brain, and especially to increase delta activity.

Obviously, further studies are needed to determine whether meditation-based approaches, such as immersive sound meditation, could have neuroprotective effects by improving glymphatic activity. If confirmed, such approaches might become increasingly important as promising non-pharmacological interventions for promoting healthy brain aging and preventing neurodegenerative disorders such as AD. From this point of view, the application of immersive sound and the installation of immersive sound environments could open new frontiers to improve brain health and enrich the practice of medicine. We therefore wish to encourage further research in this area.
